# Laryngeal tube use in out-of-hospital cardiac arrest by paramedics in Norway

**DOI:** 10.1186/1757-7241-20-84

**Published:** 2012-12-18

**Authors:** Geir A Sunde, Guttorm Brattebø, Terje Ødegården, Dag F Kjernlie, Emma Rødne, Jon-Kenneth Heltne

**Affiliations:** 1 Department of Anaesthesia and Intensive Care, Haukeland University Hospital, Bergen, Norway; 2 Department of Research and Development, Norwegian Air Ambulance Foundation, Drøbak, Norway; 3 Department of Medical Sciences, University of Bergen, Bergen, Norway; 4 Department of Medicine, Sorlandet Hospital Flekkefjord, Flekkefjord, Norway; 5 Department of Prehospital Care, Innlandet Hospital, Innlandet, Norway

**Keywords:** Out-of-hospital cardiac arrest, Intubation, Airway management, Supraglottic airway device, SGA, Laryngeal tube, LT, LTS, EMS

## Abstract

**Background:**

Although there are numerous supraglottic airway alternatives to endotracheal intubation, it remains unclear which airway technique is optimal for use in prehospital cardiac arrests. We evaluated the use of the laryngeal tube (LT) as an airway management tool among adult out-of-hospital cardiac arrest (OHCA) patients treated by our ambulance services in the Haukeland and Innlandet hospital districts.

**Methods:**

Post-resuscitation forms and data concerning airway management in 347 adult OHCA victims were retrospectively assessed with regard to LT insertion success rates, ease and speed of insertion and insertion-related problems.

**Results:**

A total of 402 insertions were performed on 347 OHCA patients. Overall, LT insertion was successful in 85.3% of the patients, with a 74.4% first-attempt success rate. In the minority of patients (n = 46, 13.3%), the LT insertion time exceeded 30 seconds. Insertion-related problems were recorded in 52.7% of the patients. Lack of respiratory sounds on auscultation (n = 100, 28.8%), problematic initial tube positioning (n = 85, 24.5%), air leakage (n = 61, 17.6%), vomitus/aspiration (n = 44, 12.7%), and tube dislocation (n = 17, 4.9%) were the most common problems reported. Insertion difficulty was graded and documented for 95.4% of the patients, with the majority of insertions assessed as being “Easy” (62.5%) or “Intermediate” (24.8%). Only 8.1% of the insertions were considered to be “Difficult”.

**Conclusions:**

We found a high number of insertion related problems, indicating that supraglottic airway devices offering promising results in manikin studies may be less reliable in real-life resuscitations. Still, we consider the laryngeal tube to be an important alternative for airway management in prehospital cardiac arrest victims.

## Background

No studies have shown significant improved survival or outcome in out-of-hospital cardiac arrest (OHCA) patients receiving prehospital intubation [1-6]. The latest European Resuscitation Council (ERC) guidelines (2010) reduce the emphasis on early endotracheal intubation (ETI) unless this intervention can be achieved by highly skilled individuals with minimal interruption of chest compressions [1].

Numerous studies have described the adverse effects associated with prehospital endotracheal intubations performed by emergency medical technicians (EMTs)/paramedics due to intubation attempts [7-11]. Also, higher incidences of difficult airways, failed laryngoscopy and factors associated with increased difficulty in obtaining an airway have been reported in the prehospital setting [12-16].

Supraglottic airway devices (SGA) have been increasingly and successfully used in resuscitation [1]. The most commonly used being the classical laryngeal mask airway (LMA), the proseal LMA, the laryngeal tube and recently the i-gel [17-19]. Several studies have shown that the SGA’s are suitable alternatives for securing the airway, both in manikins during simulated cardiac arrest and in patients during anaesthesia; however, only a few small cohort studies have described LT use in OHCA patients [20-24]. The LT is reported to be easy for inexperienced individuals and requires minimal instruction prior to first use [25-28]. Supraglottic airway devices have also shown low no-flow times and rapid airway control compared to ETI or bag mask ventilation (BMV) in cardiac arrest scenarios [22,29]. Finally, the LT is reported to be promising as a rescue device after failed ETI and in restricted patient access situations [25,30,31].

There is limited evidence regarding the optimal airway technique during cardiac arrest or which level of competence is needed for individuals managing the patient’s airway. The LT was introduced as our primary airway management tool during the initial phase of cardiopulmonary resuscitation (CPR) in Haukeland and Innlandet districts in 2002/2005. The objective of this study was to evaluate the performance of the LT in OHCA victims treated by our EMS.

## Methods

### Study setting and design

The study was designed as a dual-centre, retrospective evaluation of a single new airway device: the laryngeal tube. The recruited centers comprise two of the national health trust regions, the Haukeland University Hospital region and the Innlandet Hospital region, covering a population of around 900 000 people and 70,000 km2 (roughly half the size of England) with approximately 75 ground ambulance units. Being a retrospective study, we did not have a formal written protocol establishing the sequence of actions using the LT in OHCA. However, both participating ambulance services have similar standard operating procedures (SOP) for OHCA, stating the sequence of actions to be taken in these situations. The actual LT procedure with emphasis on minimising hands-off time during CPR, the LT was inserted directly without previous BMV, during on-going chest compressions. Successful attempt is defined as completed insertion of the LT combined with adequate ventilation of the patient assessed by inflation of the lungs, or visible chest movement, and confirmed by auscultation or capnography. If the attempt is not successful within 30 seconds, the patient has to be ventilated/oxygenated with BMV or pocket mask before a new attempt can be made. After a maximum of three LT attempts the method is abandoned, returning to BMV or pocket mask.

### EMS organization

The Norwegian EMS is organised in an uniform way where the emergency medical communication centres (EMCC) are accessed by a national three digit number (113), from which the nearest ambulance and/or primary care doctors on-call are alarmed. The EMCC use the Norwegian Index of Medical Emergencies as a decision tool for level of emergency, and dispatch health resources accordingly [32,33]. In pre-hospital cardiac arrests, the EMCC provides telephone guided CPR to lay people if the patient is unconscious with abnormal breathing. Simultaneously, the nearest ambulance/primary care doctor on-call and first-responder with automated external defibrillator (AED) are dispatched to ensure advanced life support (ALS). If available, the nearest helicopter emergency medical service (HEMS) staffed by anesthesiologist may also be dispatched.

### Ambulance personnel education and training

The ambulance system is part of the hospital organization, and all personnel are employed by the health trusts. The educational background can vary, from basic EMTs (emergency medical technicians), via paramedics to nurse anaesthetists working in the capacity of ambulance personnel in EMS, and also anesthesiologist if the air ambulance service is responding to the actual OHCA. The term”anesthesia-trained providers” refers to nurse anaesthetists and anesthesiologists.

During basic training they are introduced to the LT during their airway management module, in addition to bag-mask-ventilation. All ambulance personnel received an initial 3 hour lesson on airway management followed by supervised training on manikins with the LT. The training session was followed by a written and practical test in a manikin assessed by supervisors. The EMS personnel also had to take part in an annual formal retest, where LT use was tested in simulated cardiac arrest scenarios.

### The laryngeal tube

The LT is a SGA and comes in single lumen (LT + LT-D) or dual lumen versions (LTS II + LTS-D with suction/drain tube), both available in reusable or disposable forms (VBM Medizintechnik GmbH, Germany). The LT has 2 cuffs inflated by one syringe, sealing both the oesophagus and the oropharynx, and with ventilation holes over the laryngeal inlet. Our ambulance services have used various laryngeal tube models (LT/LTSII/LTS-D) in accordance with their enhancements, as their primary device for airway management in OHCA patients since 2002 (Haukeland) and 2005 (Innlandet). Suitable LT size was in each case chosen based on estimated patient height and according to product guidelines. LT size 3, 4 and 5 are used in adults.

### Data collection and subjects

Post-resuscitation forms and data on airway management in OHCA patients were assessed with regard to LT insertion success rates, insertion time, insertion-related problems, and the ease of insertion. 347 adult non-traumatic out-of-hospital cardiac arrest patients in our 2 regions between 2002-2010, where LT was used as airway method, were included in this study. All cardiac arrest ambulance personnel were instructed to document the related airway data variables every time of involvement. Data variables included the choice of primary airway method (mouth-mask-ventilation (MMV), bag-mask-ventilation (BMV), or laryngeal tube (LT)) and verification of correct placement (auscultation and end-tidal carbon dioxide measurement). In addition all insertion-related problems like air leakage, problematic initial tube positioning, tube dislocation, absence of respiratory sounds on auscultation, insertion time exceeding 30 seconds, and provider-rated ease of insertion (“Easy”, “Intermediate” or “Difficult”) were recorded. A provider self-reporting method was used and in our study this covered his/hers perception of the procedure difficulty as a whole, from insertion attempt to confirmation of ventilation. End tidal carbon dioxide (ETCO2) measurements were performed using the Easy Cap II CO2 Detector® (Covidien, USA) or were available on the Lifepak® 12 Defibrillator (Physio-Control Inc., USA).

### Data analysis

Microsoft Excel 2011 for Mac Version 14.0® (Microsoft Corporation, USA) and SPSS version 19.0® (IBM SPSS Inc., USA) were used for data analysis and presentation.

### Ethics

The Regional Committee for Medical Research Ethics (REK-Vest) exempted the study from ethical approval, regarding it as a quality improvement study. The ethics committee declared no objections toward the study or the publication of the results. The Data Protection Official for Research (Helse Bergen) approved the study. Permission was only given for anonymous data review, and the post-resuscitation forms and data from airway management were anonymous to personal data. As such, outcome data and demographics from EMS or in-hospital patient medical records could not be obtained.

## Results

### LT insertion success rates

**Figure 1:**
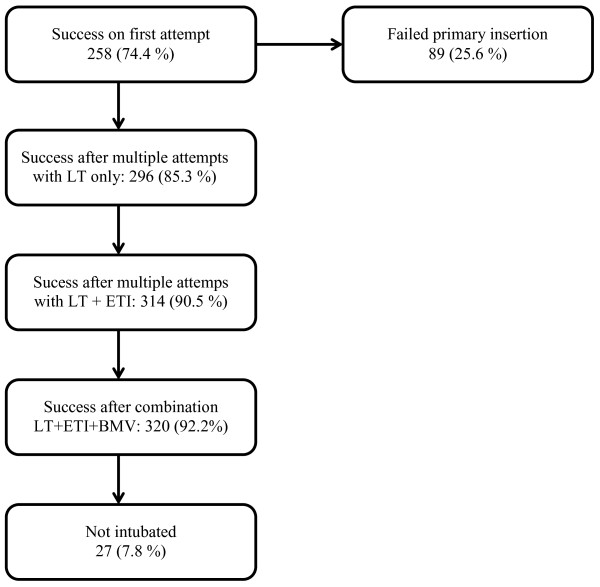
Insertion success in 347 patients.

A total of 402 insertions were performed in 347 OHCA patients. Overall, LT insertion was successful in 85.3% of patients, with a 74.4% first-attempt success rate. Insertion success is shown in Figure 1. In the 89 patients (25.6%) where primary insertion attempt failed, the reattempt airway device used was either the LT (42.7%), ETI (20.2%), BMV (6.7%), or a combination of the three (6.7%). Data reporting rescue airway technique in 21 of the primary failed insertions was missing. In 2.4% of the successful insertions, the LT was converted to ETI by anesthesia-trained providers during the resuscitation.

### Insertion time

In a minority of patients (n = 46, 13.3%), the LT insertion time exceeded 30 seconds. The mean number of insertion attempts was 1.16 (range 1-4). Insertion time exceeding 30 seconds was a result of difficult single insertions (n = 15) or multiple insertion attempts (n = 31).

### Insertion-related problems

Insertion-related problems were recorded in 52.7% of patients, as shown in Table 1.

**Table 1:** Insertion related problems with the Laryngeal tube in 347 patients

**Insertion related problems**	**No. of patients (%)**
none	164 (47.3%)
lack of breath sounds (auscultation)	100 (28.8%)
problematic initial tube positioning	85 (24.5%)
leakage	61 (17.6%)
insertion time > 30 sec	46 (13.3%)
vomit/aspiration	44 (12.7%)
tube dislocation	17 (4.9%)

### LT size and verification of placement

The following LT sizes were used in our patient population: LT size 4 (n = 208 patients), size 5 (n = 141 patients), and size 3 (n = 11 patients). The primary methods used to verify correct placement were auscultation (n = 252, 72.6%) and ETCO2 confirmation (n = 213, 61.4%). The mean age of patients was 66.8 years (SD ± 16.4 years).

### Ease of insertion

The difficulty of insertion was scored for 95.4% of the patients, with the majority of insertions being assessed as “Easy” (62.5%) or “Intermediate” (24.8%) by the airway providers. Only 8.1% of the insertions were regarded as “Difficult”.

## Discussion

Our data suggest that ambulance EMTs/paramedics in our EMS insert the laryngeal tube with acceptable success rates in adult out-of-hospital cardiac arrest patients compared to others [23,34]. We consider the laryngeal tube to be an important supraglottic alternative for airway management in prehospital cardiac arrest victims treated by EMTs/paramedics.

Endotracheal intubation has traditionally been regarded as the optimal method for securing the airway during cardiac arrest [1]. However, as shown by Wang et al, EMT and paramedic manned EMS have demonstrated low ETI success rates of between 72-82% across several patient categories, including OHCA as well as intubation-associated CPR interruptions [11,35]. The Ontario Prehospital Advanced Life Support Study (OPALS) found no improvement in OHCA survival after adding advanced life support (ALS) interventions to an emergency medical services (EMS) protocol with rapid defibrillation [36]. Also, a recent Cochrane review stated that prehospital intubation in non-traumatic cardiac arrest patients is less likely to benefit patients than early defibrillation and bystander CPR [6].

Several studies report SGA insertion success rates as being relatively independent of the rescuers’ competence levels (i.e., inexperienced providers can establish an airway as quickly and effectively as more experienced rescuers after simple airway management training) and that inexperienced providers may establish airways faster and more efficiently with SGAs (e.g., LT compared with ETI or BMV) [20,29,37]. However, in our study we found SGA insertion success rates between 74-85%, similar to that described for first-responders and comparable EMS-services in other countries, but also not much better than the ETI success rates described by Wang et al [20,34,35]. Also, as rescue device after failed primary insertions, renewed attempts with the LT were successful in only half of the cases in our study.

According to our SOP, the LT was inserted directly without previous BMV, during on-going chest compressions. Reduced no-flow times using the LT, as shown by Wiese et al, did not apply to our study [22]. After a patent airway is established, the hands-off time is independent of the actual airway device chosen. The majority of LT insertions were completed within 30 seconds, and fast insertion times have also been showed by other authors, e.g Lanimaki et al and Kurola et al [20,34].

Our study showed a relatively high number of insertion-related problems using the LT in OHCA, and to our knowledge these complications have not been described elsewhere. Among the problems described (Table 1), problematic initial tube positioning, leakage, vomit/aspiration and tube dislocation may impede effective ventilation and oxygenation of the patients. These problems were encountered in over 50% of our patients which seems high, but some of these may be of less importance. Lack of respiratory sounds (28,8%) on auscultation does not necessarily mean ineffective ventilation if chest movement is observed. How experienced the participants were in the auscultation technique, combined with a noisy prehospital environment, may influence these results in a synchronous ventilation and chest compression scenario.

Endotracheal intubation requires more extensive training than most EMTs/paramedics receive to obtain adequate skill levels, and the basic bag-mask-ventilation technique used by most EMS personnel may be difficult to perform correctly [8,24,38-41]. In earlier studies, the feasibility of establishing airway and ventilation with SGA’s has been demonstrated, and SGA’s may be associated with better skill retention compared with ETI [24,25,34]. Our data indicate that devices offering promising results in manikin studies may be less reliable in real-life resuscitations. We suggest that even the seemingly better and simpler airway solutions may demand better airway training programmes in the future, along with engineering advances in supraglottic devices.

The majority of insertions in our study were rated as easy or intermediate, with only 8,1% rated as difficult. Similar self-reporting has been used in other studies [34]. However, the self reporting of airway management difficulty was in contrast to the amount of insertion related problems experienced, and we believe this may be due to unreliability in self-reporting and the level of provider competence in our study.

The limitations of our study include its retrospective methodology and the evaluation of a single airway device. Limitations in data completeness and compliance issues have also been noted in other reviews of EMS patient records, and retrospective chart reviews can miss critical events, e.g., misplaced tubes due to observation bias and underreporting [42]. Also, there are no golden standards for measuring procedural difficulties in healthcare, and provider self-reporting may be prone to bias. Furthermore, our study includes only a limited series of adult OHCA victims; thus, the results may not be generalisable to other patients.

Nevertheless, being a dual-centre study and among the first to evaluate the performance of EMTs/paramedics with the LT in nearly 350 OHCA patients, we believe that our study offers new information regarding airway management in these patients; e.g. the insertion related problems recorded. Future studies comparing advanced versus basic airway techniques in adult and paediatric cardiac arrest victims are needed, and the continued development of improved supraglottic devices for EMS use are encouraged.

## Conclusions

Our study suggests that ambulance emergency medical technicians (EMTs) and paramedics in Norwegian emergency medical services (EMSs) insert laryngeal tubes with acceptable success rates in adult OCHA victims. We found a relative high number of insertion related problems, indicating that supraglottic devices offering promising results in manikin studies may be less reliable in real-life resuscitations. Still, we consider the laryngeal tube to be an important alternative for airway management in prehospital cardiac arrest victims.

## Abbreviations

LT: Laryngeal tube; OHCA: Out-of-hospital cardiac arrest; EMT: Emergency medical technician(s); EMS: Emergency Medical Service(s); ERC: European Resuscitation Council; ETI: Endotracheal intubation; SGAs: Supraglottic airway device; LMA: Laryngeal mask airway; BMV: Bag-mask ventilation; CPR: Cardiopulmonary resuscitation; SOP: Standard operating procedure; EMCC: Emergency medical communications centre; AED: Automated external defibrillator; ALS: Advanced life support; HEMS: Helicopter emergency medical service; MMV: Mouth-mask ventilation; ETCO2: End tidal carbon dioxide.

## Competing interests

The authors declare that they have no competing interests.

## Authors' contributions

GAS, GB and JKH conceptualised the study and participated in its design and coordination; they were also instrumental in drafting the manuscript. ER participated in the design of the study, sampling of the data, and drafting of the manuscript. DFK and TØ participated in the collection of the data and the drafting of the manuscript. All authors have read and approved the final version of the manuscript.

## Author information

The authors are employed by Haukeland University Hospital, Innlandet Hospital or Sørlandet Hospital Flekkefjord. All hospitals are a part of the national health system and are funded by the Norwegian government. GAS is also employed at the Norwegian Air Ambulance Foundation. The study received no external financial support or grants.
